# Levonorgestrel correlates with less weight gain than other progestins during hormonal replacement therapy in Turner Syndrome patients

**DOI:** 10.1038/s41598-020-64992-4

**Published:** 2020-05-19

**Authors:** Andréia Latanza Gomes Mathez, Patrícia Teófilo Monteagudo, Ieda Therezinha do Nascimento Verreschi, Magnus Régios Dias-da-Silva

**Affiliations:** 1 Endocrinology Division, Department of Medicine, Escola Paulista de Medicina, Universidade Federal de Sao Paulo, Sao Paulo, Brazil; 2 Laboratory of Molecular and Translational Endocrinology, Escola Paulista de Medicina, Universidade Federal de Sao Paulo, Sao Paulo, Brazil

**Keywords:** Endocrine reproductive disorders, Endocrine reproductive disorders, Hormonal therapies, Hormonal therapies

## Abstract

Turner Syndrome (TS) is associated with an increased risk of cardiovascular and metabolic complications. Furthermore, TS women need hormone replacement therapy (HRT), of which progestins can influence body weight. We aimed to analyze the metabolic and weight profile in a cohort of 111 TS women. They started receiving estrogen at 15.8 (±3.6) years old, with no change in hypertension, dysglycemia, and dyslipidemia incidence but with a tendency to increase overweight (*p* = *0.054)*. As the first used type of progestin, most had received cycles of 10 days per month of medroxyprogesterone (MPA) or levonorgestrel (LNG), then shifted to micronized progesterone (MP), which has currently become the most used one. By multiple linear regression analysis, we found that the prolonged use of MPA, LNG, or MP showed no metabolic change except for weight gain. The percentage of annual BMI increment was positive for all progestins used in TS women (MPA 2.2 ± 2.2; LNG 0.2 ± 1.2; and MP 2.2 ± 2.6 kg/m2), but LNG seemed to best prevent on weight gain over time (*p* < *0.05)*. In conclusion, metabolic comorbidities are prevalent in TS even before the HRT regimen, and LNG performed better on less weight gain than MPA and MP in our cohort of the TS population.

## Introduction

Turner syndrome (TS) is a chromosomal sex disease that affects 1:2.500–1:4.000 females. It is characterized genetically by missing or structurally altered one of the X chromosomes, and clinically by ovarian failure, infertility, and short stature. TS diagnosis is confirmed by the typical karyotype 45,X, or partial monosomy with or without mosaicism^1–4^. The X chromosome monosomy incurs in gonadal dysgenesis, comprising 85% of patients with primary amenorrhea and 98% infertility. Although Turner girls may present complete puberty spontaneously in 15%^2^, breast development may reach 48%^5^. TS adolescents present marked hypergonadotropic hypogonadism, so puberty development usually needs pharmacological induction at about the age of 12^3^.

TS *per se* is associated with a three to fourfold increase in mortality related to cardiovascular complications and increased risk of metabolic diseases such as dysglycemia, dyslipidemia, hypertension, and obesity^2,4,6^. Hypertension affects up to 60% of this population, and there is a type 2 diabetes (T2D) risk increase in two to four times. The body mass index (BMI) is higher than the general population not only because of the short stature but also related to more visceral adiposity. Furthermore, some studies found an increased incidence of hypertriglyceridemia, low HDL, elevated and more atherogenic LDL, independently of being already obese^1,2,4^. Nonalcoholic fatty liver disease (NAFLD) is often found, and it is related to insulin resistance (IR)^7^.

Many factors have been studied as possible influences for developing metabolic comorbidities. Hypogonadism is a factor that contributes to the increase in metabolic diseases in TS women. Besides, it is associated with low bone mineral density and psychological variations^4^. Hypothyroidism is the most frequent autoimmune disease affecting this population, but it does not seem to modify the lipid profile or BMI^8^. Also, the treatment with somatotropin improves stature without changing corporal composition, independently of dose, duration, or age at onset of the therapy’s beginning^9^.

The hypogonadism treatment is based on hormonal replacement therapy with estrogen (E) plus progestin (P). It is well known that E is essential not only for puberty induction and maintenance but also for bone mass gain and metabolic improvement^10^. Both oral and transdermal routes have the same effect over corporal composition, blood pressure, hepatic enzymes, bones, carbohydrate metabolism, and lipidic profile, except for one study reporting higher HDL after taking oral estradiol^1,11^. Transdermal estradiol (TE2) has shown to be the most appropriate E for replacement therapy and its pharmacokinetics prevents hepatic first-pass and achieves physiological E2 serum concentration with a lower risk of thrombosis^1,3,10,11^.

Progestins input should start around two years after estrogen replacement or after the first induced menses^3^. However, progestins may have effects on androgenic, glucocorticoid, and mineralocorticoid receptors other than progesterone receptor (PR)^11^. As a result, the continuous use of progestin (P), substantially present in contraceptive pills, more markedly when with medroxyprogesterone acetate (MPA), is associated with weight gain and decreases in lean mass^12,13^.

Therefore, it is of great importance to ensure that the hormone replacement therapy (HRT) offered to TS women is no longer a factor of cardiometabolic risk increase as it is going to be used for a long time, at least until the usual menopause age of general population^1,3,14,15^.

Based on this, we performed a retrospective study to analyze the correlation between TS’s metabolic profile and the use of oral (P), and to examine the best therapeutic alternative to be employed in HRT in this population. The hypothesis addressed in this study is that MPA would contribute to the worsening of metabolic risks in TS patients, given that it also has glucocorticoid activity.

## Patients and Methods

### Patients

We performed a cross-sectional and non-controlled retrospective study of the TS cohort followed at the Endocrinology Outpatient Clinics of Hospital São Paulo from the Universidade Federal de São Paulo. All of them have a diagnosis based on the clinical profile and karyotype. They signed the informed consent to participate in this study after the protocol was properly approved by the Ethics Committee in Research (research number 1146/2015). All methods were performed in accordance with the relevant guidelines and regulations.

All enrolled TS patients were on HRT. Of note, not having an ideal control group is justified because of the limitation of having TS adults who had not received HRT, which of course it would be non-ethical. We excluded TS patients who had used norethisterone, gestodene, cyproterone acetate, and drospirenone as contraceptive pills prescribed by other services.

We evaluated retrospectively 123 medical records from TS patients that started HRT. However, 12 of them were excluded due to inconsistent clinical information (addressed from other services), irregular follow-up, still on growth hormone or non-compliancy with levothyroxine (LT4) therapy. This cross-sectional analysis comprises Phase 1, with which we identified general clinical information regarding the percentage of 45,X karyotype, current age in years, if hypothyroid on regular treatment, GH therapy before the start of HRT, metabolic data (anthropometric, hypertension, lipids profile, glycemic measurements), and the age at estrogen and progestin onset.

We further stratified them in three other retrospective analytical phases. Phase 2.1 comprised those from the very beginning of estrogen introduction until the start of the progestin association. Then, we only considered patients in regular HRT and with documented estrogen and progestin start time to be able to precisely evaluate the type and age at HRT onset used to pubertal induction and the time of the last outpatient follow-up visit.

**Figure 1:**
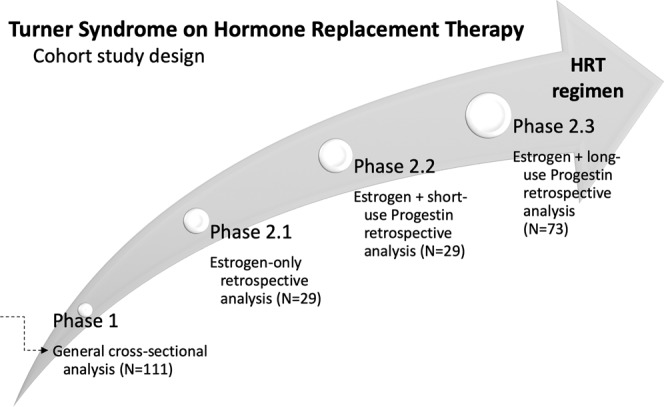
Schematic overview of our study regarding hormone replacement therapy regimen used for Turner Syndrome patients. Patients enrolled in each phase analysis with the criteria for including and excluding Turner Syndrome patients. For general cross-sectional analysis, we invited 123 TS patients, but 12 were excluded due to uncontrolled metabolic morbidities, irregular follow-up, still on GH, hypothyroid, no HRT compliance, and no wanted to participate (N = 111); for estrogen-only retrospective analysis, we selected TS patients on estrogen with no other uncontrolled metabolic comorbidities (euthyroid on regular LT4, no GH), incomplete metabolic data at estrogen start (N = 29); for estrogen + short-use progestin retrospective analysis, we enrolled those TS on estrogen and within the 1st year of progestin with no other uncontrolled metabolic comorbidities, and excluded those due to incomplete metabolic data at progestin start or the end of the 1-year follow-up (N = 29); For estrogen + long-use progestin retrospective analysis, we evaluated TS women on estrogen and over one year of progestin (long-use) with no other uncontrolled metabolic comorbidities and complete metabolic data at progestin start (N = 73). For BMI annual increment study, we evaluated 37/73 in regular use of MPA, LGN, and MP.

Moreover, we selected those to study the progestin effect on metabolic comorbidities over time, being Phases 2.2 and 2.3 the short and long use of progestin, respectively. Patients who had changed their type of progestin entered Phase 2.2 and Phase 2.3 study more than once. Figure 1 describes the number and characteristics of TS patients enrolled in each analysis. For the Phase 2.2, characterized by HRT with estrogen and within the first year of progestin, we included those patients with available metabolic data from the last consultation before using progestin and exactly 1 year after use. For Phase 2.3, the long retrospective analysis of estrogen and progestin HRT, we included those patients in regular HRT with available metabolic data from the time just before starting progestin until the last examination. TS patients without progestin or less than one year use, in irregular use, or with any medical condition or medication, such as corticosteroids or insulin, which could have interfered with metabolic parameters, were excluded.

### Clinical and lab evaluation

All data were collected from the medical records including karyotype, age at which estrogen and progestin started, type of estrogen used to induce puberty and at the last medical visit, whether progestin was changed, and what had been used until last outpatient visit, the period of time between estrogen and progestin introduction, time of progestin exposure (total and for each progestin used), and metabolic parameters before HRT, after 1 year of each progestin, and longtime use based on the last follow-up visit. For each situation, clinical parameters were evaluated by measuring the weight and height for body mass index (BMI) calculation (weight square of height) or for the BMI z-score (Standard Deviation Score) if under 20 yo; also the systolic blood pressure (SBP) and diastolic blood pressure (DBP) were noted. The z-score was calculated with the WHO app, Anthro-Plus (http://www.who.int/childgrowth/software/en/). Laboratory data were retrieved for fasting glycemia (FG), triglycerides (TG), high-density lipoprotein (HDL), low- density lipoprotein (LDL), TSH, and renal function.

We followed standard altered clinical and lab metabolic parameters as defined: hypertension when using of antihypertensive drugs or SBP ≥ 140 mmHg or DBP ≥ 90 mmHg in adults or if greater than 95^th^ Percentile when age under 20yo [9); overweight when BMI > 25Kg/m² in adults or z-BMI > 1 if age inferior to 20yo; obesity was defined as BMI ≥ 30 kg/m^2^ in adult patients or if standard deviation score body mass index (z-BMI) ≥ 2 if age inferior to 20yo; dysglycemia with pre-diabetes when FG 100–125 mg/dl or HbA1C 5.7–6.4%, or diabetes when FG ≥ 126 mg/dl or HbA1C ≥ 6.5% or in use of an oral antidiabetic [10); dyslipidemia when HDL-C < 50 mg/dl (HDL < 45 before 19 years old), LDL-C ≥ 160 mg/dl, TG ≥ 150 mg/dl (TG ≥ 130 before 19 years old) or in statin use (13).

### Progestin use evaluation

Since TS patients used more than one progestin in different periods of the HRT follow-up, either due to health system availability, personnel or lack of family financial resources, and adverse side effects, we have developed a method to combine and normalize the period and the dose of progestin use by creating a unit for measuring the progestin exposure by the Standard Progestin Cumulative Dose (SPCD).

We applied SPCD rule to normaize combined and interposed most common oral progestins used in each HRT for 10-day-per-month cycles within one year: Medroxyprogesterone Acetate (MPA), Levonorgestrel (LNG) and Micronized Progesterone (MP). Also, we assumed that the daily dose, 10 days of use per month, and years of use have equal influence as progestogen effect. One SPCD unit for each MPA (5 mg/day × 10 days per month × 1 year = 5 mg/10d/yr.), LNG (250 mg/day × 10 days per month × 1 year = 250 mg/10d/yr.), and MP (100 mg/day × 10 days per month × 1 year = 100 mg/10d/yr.) was considered equalized. As a result, a hypothetical patient who used MPA on the scheme of 10 mg/10d/month for 1 yr has an SPCD of 2, which was the same as a patient who has 10 mg/5d/month for 2 yr, LNG 0.25 mg/10d/month for 2 years, or MP 200 mg/10d/month for 1 year.

### Statistical analysis

Data were presented as Mean ± Standard Deviation for numeric parametric or Median (Minimum-Maximum) for non-parametric variables and by number and percentage for qualitative variables. Shapiro Wilk test was applied to check for normal population distribution, with equal variances among individuals (p-value 0.05). A comparison of means was performed using Student’s paired t-test or One Way ANOVA complemented with Bonferroni’s Test for all pairwise comparison, when there was normal data distribution. If not, the Student’s unpaired t-test, or Repeated Measures ANOVA on Ranks (Kruskal-Wallis on Ranks) complemented with Dunn’s Method for all pairwise comparison was used, and Spearman correlation for non-parametric distributions accordingly. Multiple Linear Regression was applied for annual variation in body mass index relative to pre-progestin BMI versus type of progestin. Wilcoxon Signed Rank Test was applied for before and after treatments comparison. The Contingency Tables were used to determine whether or not the distribution of each group was contingent on the categories it fell in, and the Fisher Exact Test was applied. Yates Correction Factor was used for a more accurately computed p-value in the significant cases. A significance level of 0.05 was adopted for all analyses. The power considered was ‘greater than 0.80’. The analyses were performed using the software’s Sigma Plot 13.2 for Windows (Systat Software Inc.), Statistical Package for Social Sciences (SPSS version 24), Minitab 16, and depicted in Excel Office 2010.

## Results

### General TS cohort features on hormone replacement therapy (HRT)

At the time of puberty induction, based on 73 TS patients with pediatrics available documentation, most TS patients (45/73; 62%) received conjugated equine estrogens (CEE), followed by estradiol valerate (VE2). Nowadays, among 111 patients using estrogen replacement, 56 (50%) are using transdermal 17β-estradiol (TE2), 51 (46.4%) VE2, and 4 (3.6%) Ethinyl estradiol (EE2). Regarding the type of progestin, 62/73 (56%) used medroxyprogesterone acetate (MPA) at the beginning, but currently, its use was reduced to 6/111 (9%). About 54/111 (49%) are using levonorgestrel (LNG), and 41/111 (41%) micronized progesterone (MP). All the enrolled patients have used oral (OE2), or transdermal E2 (TE2) with different progestins for HRT, being most with MPA, LNG, and MP. General clinical data are shown in Table 1.

**Table 1:** Overall clinical features of the cohort at basal analysis -Phase 1.

Clinical feature	Number of subjects
45, X Karyotype (%)Non-45, X, n (%)	79/111 (71.2)32/111 (28.8)
Current age in years	25.7 ± 12.2
Hypothyroidism on regular treatment	29/111 (26.1)
GH therapy before HRT	75/111 (67.6)
Age at estrogen onset in years (range)	15.8 ± 3.6 (9.6–29.2)
Age at progestin onset in years (range)	18.2 ± 3.7 (12,5–29.6)
Time between estrogen and progestin onset, in years	2.17 ± 2.1

Karyotype distribution, age (mean ± SD), the prevalence of thyroid disfunction, GH and HRT use in the 111 girls with Turner syndrome. Data showed either as mean ± SD or N/total (%).

### Metabolic changes on HRT

We assessed metabolic changes observed in TS patients regarding the impact of HRT. The objective here was to verify if progestin onset would interfere in the metabolic parameters such as hypertension, dysglycemia, dyslipidemia, and weight gain. None of them were smokers or alcohol users, neither using any drug influencing blood glucose, blood pressure (BP), lipid profile (except when the treatment of these conditions was necessary), and weight. Twenty-four obese TS patients underwent either an abdominal ultrasound or hepatic biopsy, and 8/24 (33%) presented with hepatic steatosis.

We accessed the precise period from the beginning of estrogen replacement until the progestin association based on medical record information available for 29 patients, justifying the number of cases enrolled for Phase 2.1 study, as shown in Fig. 1 and Table 2B. Also, data from 29 patients were available for the first year of the association of progestin (Phase 2.2, Table 2C). For Phase 2.3, we were able to select 73 TS women with a full lab and clinical documentation. It is of note that the number of patients with complete data varied regarding each metabolic feature. The proportion of overweight plus obese increased significantly with estrogen plus progestin HRT. When initially comparing the baseline and after taking estrogen-only, observed a trend in increased weight (*p* = *0,054*) (Table 2A vs. 2B). We found a significantly higher frequency of overweight/obesity when we compared TS patients before and after prolonged progestin use in HRT (Table 2A vs. 2D). Although the rates of hypertension, dysglycemia, and dyslipidemia did not significantly change, we observed that TS patients tended to present more hypertension (*p* = *0.071*) (Table 2D). Besides we were able to demonstrate a significant correlation of SPCD with improving HDL level (rs 0.38; *p* = *0.04*) and worsening BMI (rs −0.32; *p* = *0.01*). SPCD for each evaluated progestin (MPA, LNG, and MP) correlated positively with BMI (rs 0.30; *p* = *0.0*2).

**Table 2:** Prevalence of metabolic changes observed in Turner Syndrome patients along with different hormone replacement therapy regimens.

Metabolic changes	N (total)		%	*P*-value
A-Before HRT				
Hypertension	4 (38)		10.5	
Dysglycemia	3 (28)		10.7	
Dyslipidemia	8 (23)		34.8	
Overweight/ Obesity	10 (43)		23.2	
B-HRT with estrogen-only				*A vs B*
Hypertension	6 (28)		20.7	*0*.2*22*
Dysglycemia	2 (13)		15.4	*0.670*
Dyslipidemia	6 (12)		50.0	*0.383*
Overweight/ Obesity	13 (29)		44.8	*0.054*
C-HRT with estrogen and within the first year of progestin				*A vs C*
Hypertension	3 (25)		12.0	*0.855*
Dysglycemia	0 (19)		0	*-*
Dyslipidemia	9 (24)		37.5	*0.846*
Overweight/ Obesity	12 (29)		41.3	*0.101*
D-HRT with estrogen and after long-term of progestin				*A vs D*
Time evaluated (years)*	2.67 y (1.00 to 14.08 y)			
Hypertension	15 (59)		25.4	*0.071*
Dysglycemia	6 (59)		10.2	*0.937*
Dyslipidemia	18 (66)		27.3	*0.495*
Overweight/Obesity	43 (73)		58.9	0.00019*

A and B, Phase 2.1, HRT before and after estrogen-only; C, Phase 2.2, HRT with estrogen and within one year of progestin. D, Phase 2.3, HRT with estrogen and long-term progestin. HRT, Hormone Replacement Therapy, *time evaluated as the average and minimum-maximum. In parenthesis is represented the number of patients with available data regarding that aspect analyzed.

Since some patients had used more than one type of progestin during the follow-up, they were analyzed more than once considering, the SPCD before and after each progestin applied in the HRT, being 57 TS patients in an overlapped analysis. Some patients using MP nowadays were those who had used either MPA or LNG in the past. They presented similar results in lab tests such as HbA1C, FG, HDL, LDL, and TG. There was no significant change in these parameters after one year-use of each SPCD.

### Weight gain and the type of used progestin

Initially, we studied the weight change of 73 TS patients with a mean age of 22.0 ± 6.3 yo. regardless of the type of used progestin in HRT. Then, we were able to select 61/73 TS patients who had information of the precise period of each type of progestin, in an attempt to stratify the analysis per subtype of progestin concerning its influence in weight gain. We found that using MPA increased weight more than LNG or MP (3.9 ± 5.81 vs. 3.0 ± 2.75 and 1.5 ± 2.24, respectively; p < 0.05). However, evaluating only young TS patients 24/73 (<19 yo.), with a mean age of 16.6 ± 1.41 yo, using progestin for 3.6 ± 2.9 years (from 1 to 8.8 y.), there was no significant change in z-BMI scores (Mi − 0.04, from −0.79 to 3.50). In this young subgroup, 16 were taking MPA, 3 LNG and 5 MP, and comparing the rate of z-BMI change for each type of P (MPA vs. LNG and MP; Wilcoxon Signed Test), there was no different evolution between them.

**Figure 2:**
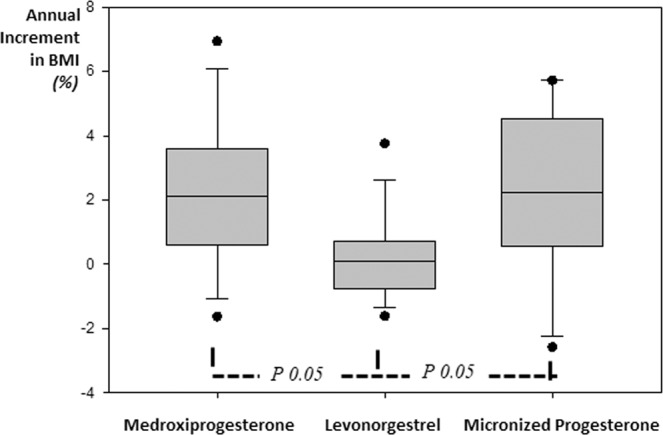
Graphic representation of the annual increment in body mass index accordingly with progestin use. We evaluated the differences in mean values among groups. *p* < *0.05* value, when increment is greater than it would be expected by chance. MPA medroxyprogesterone, LNG levonorgestrel, MP micronized progesterone. SPCD, standard progestin cumulative dose. BMI body mass index.

When analyzing only the TS adult (>19 yo) group (n = 37/61), with a mean age of 25.8 ± 5.8 yo, who had used progestin for 3.6 ± 2.5 years (from 1 to 8.9 y), those using MPA were significantly younger than MP ones, so they were more exposed to the progestin as shown in Table 3. We corrected this bias in body weight change for the time evaluated, dividing weight variation by the years of exposition. We then observed that the mean annual percentage variation on BMI was significantly lower with LNG than those who had used MPA or MP. Weight gain with MPA and MP was similar, as shown in Fig. 2.

**Table 3:** Body mass index variation in adult Turner Syndrome patients stratified by the types of progestin used in the hormone replacement therapy.

Progestin	N	Age*yo*.	Time*yr*.	SPCD	Initial BMI*Kg/m*^*2*^	After BMI*Kg/m*^*2*^
*MPA*	12	22.6 ± 3.6*	5.5 ± 2.0*	6.0*(1.0 to 11.9)	22.9 ± 4.1*	25.5 ± 5.9
*LGN*	14	25.0 ± 6.1	3.4 ± 1.9	2,8(1.3 to 7.5)	24.5 ± 4.4**	24.9 ± 4.3
*MP*	11	29.3 ± 5.4	1.8 ± 0.7	1,6(1.1 to 4.3)	28.7 ± 4.0	29.7 ± 4.2
*Total*	37	25.5 ± 5.8	3.6 ± 2.5	2.6(1.0 to 11.9)	25.3 ± 4.7	26.5 ± 5.2

Data expressed as mean ± SD for parametric and as median – minimum/maximum for nonparametric data distribution by different types of progestins during the long-retrospective assessment. This subgroup comprises 37/73 TS adult patients enrolled in Phase 2.3.

MPA medroxyprogesterone, LNG levonorgestrel. MP micronized progesterone; SPCD standard progestin cumulative dose; BMI body mass index. Differences in mean values among the groups are greater than would be expected by chance (p < 0,05); *MPA vs MP, **LNG vs MP.

The association between the type of progestin and weight change was confirmed by using a multiple linear regression model. Fot that, we applyied the annual percentual variation in BMI as the dependent variable and logistic variables LNG, age, duration of treatment, and cumulated exposition (SPCD) as independent ones [annual BMI variation = 0,764 + (0,600 * LNG y/n) − (0,0262 * age) − (0,0172 * duration of treatment) − (0,0144 * SCPD) (r^2^ = 0,167; adjusted r^2^ = 0,059; p = 0,029 and Normality Test Passed (p = 0,708), Constant Variance Test: Passed (p = 0,397) and power of performed test with alpha = 0,050: 0,80.

## Discussion

Our study showed, for the first time, that neither the use of estrogen alone nor ten-day monthly use of progestin replacement, independently of its type, during the first year of HRT, has modified any metabolic parameters in TS. However, we found a significant effect concerning a type of progestin for weight gain when used for a long time. The HRT with LNG showed a significantly less weight gain than with MPA and natural MP, beyond being better for weight maintenance.

According to Trolle *et al*.^16^, most treatment recommendations directed to the TS population are based on expert opinion and not evidence-based. Since TS implies in increased metabolic risk *per se*^3,17,18^ and increasing age aggravates the risk for metabolic comorbidities^19^, revisiting the long-term HRT with estrogen and progestin, mimicking the female physiological pattern the from pharmacological puberty induction until the age of menopause^2^, it is of great importance to look for a safer sex steroid treatment for them. Considering that estrogenization can improve the metabolic profile^3,17,20,21^, and it is not related to weight gain independently of the route of administration^1,11,22^, our cohort was not divided per subtype of used estrogen. We found no significant weight alteration during estrogen replacement alone. As shown by Baldin *et al*.^9^, the use of growth hormones also did not change body composition, so our group was also not divided by previous use or not of GH. All TS patients were euthyroid even though some on levothyroxine replacement, which prevented us from separate them in a different group for thyroid status.

The benefits of TE2 replacement are better than with EE2 or even oral E2, as consolidated by literature^2,17^, so we also prefer this subtype and route of E to start puberty with low-dose and gradual increases^1,2,11^. Likewise, when possible, and during follow-up, we changed the oral to transdermal route to decrease thromboembolic and stroke risk^1,11,17^. Skin allergy problems or financial constraints are the reasons for those patients still in use of oral E2. We do not advocate for the use of EE2 as HRT in TS patients.

As far as we know, no published work accessed the cyclical replacement of progestin effect over metabolic parameters, neither in normal nor in the Turner population. The main articles related to this topic address contraception, breast cancer, polycystic ovary syndrome, menopause women or animal models, situations related to daily progestin use, different from the 10-day per month regimen. TS is an interesting population to be studied concerning HRT effects once they are free from their ovarian hormones due to primary ovarian failure^23^. We focused our study on the progestin influence since previous studies showed no difference in metabolic variables with estrogens^1,11^.

As progestin is used in HRT only for an average of 10 days per month but for so many years, it is essential to consider that it may have a cumulative effect on the metabolic health, especially in TS women, since it totalizes a huge P exposure time along with the basal risk for metabolic comorbidities. Our study is the first to investigate the metabolic effect of three types of P for HRT in TS. To overcome the bias of overlapping P exposure, we have created a standardizing tool (SPCD) to measure the cumulative exposure to different P. We noted that SPCD was higher for MPA than MP and LNG. We hypothesized that MPA worsens the metabolic risk of TS women based on its cumulative glucocorticoid activity^22^.

It is well established in cell cultures that MPA has glucocorticoid properties^24^. Besides, in ovariectomized female mice, MPA produced a pro-thrombotic effect^25^. Arias-Loza *et al*. have also found that MPA in animal model abrogates protective vascular effects of 17β-estradiol because it acts not only on PR but also on AR and GR^15^. Otto *et al*. compared MPA with Drospirenone, a synthetic progesterone hormone, and found that mitogenic activity of MPA on the mammary gland is higher than drospirenone and possibly because of its greater glucocorticoid activity^26^.

In humans, MPA effects on weight were studied in the context of contraceptive therapy^27,28^. Yancey and Raleigh found evidence that progestin-only contraceptives cause weight gain with an increase in fat deposition and decreases in lean mass^28^. Regarding P and changing metabolic profile, Ozdemir *et al*., studied the effect of MPA in women with polycystic ovary syndrome and found that there were no changes in carbohydrate or lipid metabolism^29^. Moreover, P has different effects on human breast cancer because of its pharmacological ductal growth properties. Comparing MPA, natural MP, Drospirenone, and Nestorone, Fu *et al*., concluded that all of them enhanced breast cell migrations and invasion when PR immunostaining is positive (PR+)^30^. They found that the spectrum of action of P over other targets on GR, MR, and AR makes MPA the most potent on the progression of PR + breast cancer^30^.

Considering the evidence that MPA has higher action over GR than LNG and natural MP^14^, the vast majority of our patients are nowadays on MP or LNG. In our study, we found that the prolonged use of P has an impact on weight gain without worsening blood pressure, lipidic, and glycemic profile. Meanwhile, as the SPCD of the three studied progestins showed correlation with BMI over time, we proceed to investigate the effect of MPA, LNG, and MP on worsening weight.

During the estrogen and long-use of progestin HRT assessment (phase 2.3), all types of progestins increased BMI. However, no marked difference occurred in the young group, even correcting BMI for z-score, being only evident the BMI change among adult TS patients. Also, MPA impact was more prominent than with LNG and MP. Notably, we also verified that the weight gain was clearly lower with LNG than MPA, as expected due to its less glucocorticoid activity, and surprisingly, TS patients on HRT with MP also gained more weight over time similar to MPA.

Studies assessing LNG and weight gain were all in the field of contraception or menopausal hormone therapy. Modesto *et al*. compared depot-MPA, LNG-IUD, and TCu380A IUD action on weight and found that all of them increased weight after the first year and after ten years, but LNG and TCu380A devices gained less weight than depot-MPA^31^. Besides, a systematic review and meta-analysis evaluating thromboembolism found that combined oral contraceptives containing LNG have less risk for venous thromboembolism^32^, as primarily demonstrated in the Danish cohort study^33^.

Concerning natural MP, a 3-month study from Casanova and Spritzer did not found that cyclic vaginal MP alter weight or other metabolic variables in the context of menopause period^34^. Nevertheless, literature is scarce in MP study related to metabolic parameters, especially in evaluating of TS population. Although our study was carefully conducted, there were some limitations. The lack of baseline clinical and lab data from the beginning of HRT registered in patient’s medical records and in addressing letter from pediatric services to our adult unit reduced a lot our number of samples. Also, the retrospective nature of the study cannot provide a cause-effect relationship and make it difficult to assess the correlation of each P since patients used more than one type of P onwards. We reinforce that the majority of our TS patients had used MPA for some period, and they present an inherent risk for metabolic disorders when compared to the general female population^35^.

In conclusion, metabolic morbidities are prevalent in TS even before HRT and LNG seems to be a better progestin choice in comparison with MPA and MP. Prolonged HRT with LNG may have a positive impact on metabolic features. Although, LNG did not impair blood pressure, lipid and glycemic profile, and correlated with less weight gain in our study, further research is needed to indicate a recommendation.

## Data Availability

All raw data are available for further analysis upon request to corresponding author.
