# Author Correction: Levonorgestrel correlates with less weight gain than other progestins during hormonal replacement therapy in Turner Syndrome patients

**DOI:** 10.1038/s41598-020-69839-6

**Published:** 2020-07-28

**Authors:** Andréia Latanza Gomes Mathez, Patrícia Teófilo Monteagudo, Ieda Therezinha do Nascimento Verreschi, Magnus Régios Dias-da-Silva

**Affiliations:** 1 Endocrinology Division, Department of Medicine, Escola Paulista de Medicina, Universidade Federal de Sao Paulo, Sao Paulo, Brazil; 2 Laboratory of Molecular and Translational Endocrinology, Escola Paulista de Medicina, Universidade Federal de Sao Paulo, Sao Paulo, Brazil

Correction to: *Scientific Reports* https://doi.org/10.1038/s41598-020-64992-4, published online 19 May 2020

The Acknowledgements section in this Article is incomplete.

“The authors would like to thank CAPES - Coordenação de Aperfeiçoamento de Pessoal de Nível Superior - for financial support and schorlarship for Andrea Latanza Gomes Mathez, and FAPESP for research funding.”

should read:

“The authors would like to thank CAPES - Coordenação de Aperfeiçoamento de Pessoal de Nível Superior - for financial support and scholarship for Andrea Latanza Gomes Mathez, and FAPESP (14/06597-2) for research funding.”

